# Identification of viral and bacterial pathogens from hospitalized children with severe acute respiratory illness in Lusaka, Zambia, 2011–2012: a cross-sectional study

**DOI:** 10.1186/s12879-015-0779-1

**Published:** 2015-02-12

**Authors:** Paul Simusika, Allen C Bateman, Andros Theo, Geoffrey Kwenda, Christine Mfula, Edward Chentulo, Mwaka Monze

**Affiliations:** Virology Laboratory, University Teaching Hospital, RW1X Lusaka, Zambia; Centre for Infectious Disease Research in Zambia, 34681 Lusaka, Zambia; Department of Biomedical Sciences, School of Medicine, University of Zambia, P.O. Box 50110, Lusaka, Zambia

**Keywords:** PCR, Respiratory pathogen, Severe acute respiratory illness, Zambia

## Abstract

**Background:**

Morbidity and mortality from respiratory infections are higher in resource-limited countries than developed countries, but limited studies have been conducted in resource-limited settings to examine pathogens from patients with acute respiratory infections. Influenza surveillance has been conducted in Zambia since 2008; however, only 4.3% of patients enrolled in 2011–2012 were positive for influenza. Therefore, we examined non-influenza respiratory pathogens in children with severe acute respiratory illness (SARI) in Zambia, to estimate the scope of disease burden and determine commonly-identified respiratory pathogens.

**Methods:**

Two reverse transcriptase polymerase chain reaction (rRT-PCR) methods (single and multiplex) were used to analyze nasopharyngeal and throat swabs collected from SARI cases under five years of age from January 2011 through December 2012. All specimens were negative for influenza by rRT-PCR. The panel of singleplex reactions targeted seven viruses, while the multiplex assay targeted thirty-three bacteria, fungi, and viruses.

**Results:**

A set of 297 specimens were tested by singleplex rRT-PCR, and a different set of 199 were tested by multiplex rRT-PCR. Using the singleplex assay, 184/297 (61.9%) specimens were positive for one or more viruses. The most prevalent viruses were human rhinovirus (57/297; 19.2%), human adenovirus (50/297; 16.8%), and respiratory syncytial virus (RSV) (45/297; 15.2%).

Using multiplex PCR, at least one virus was detected from 167/199 (83.9%) specimens, and at least one bacteria was detected from 197/199 (99.0%) specimens. Cytomegalovirus (415/199; 208.5%) and RSV (67/199; 33.7%) were the most commonly detected viruses, while *Streptococcus pneumonie* (109/199; 54.8%) and *Moraxella catarrhalis* (92/199; 46.2%) were the most commonly detected bacteria.

**Conclusions:**

Single infections and co-infections of many viruses and bacteria were identified in children with SARI. These results provide an estimate of the prevalence of infection and show which respiratory pathogens are commonly identified in patients. Further studies should investigate causal associations between individual pathogens and SARI.

## Background

The World Health Organization (WHO) estimates that 20% of hospitalizations in children under 5 years old are attributable to severe acute respiratory illness (SARI), and 90% of these illnesses are a result of pneumonia [1]. Furthermore, the WHO estimates that acute respiratory infections account for 1.9 to 2.2 million childhood deaths annually, with 42% occurring in Africa [2,3]. Bacterial infections, especially those that cause pneumonia, are important because they substantially contribute to childhood mortality [4-7]. Viruses also play a role, because primary infection with viral pathogens can pre-dispose children to secondary bacterial infection [8-10], and a viral diagnosis can minimize the overuse of antibiotics [11]. Furthermore, co-infection with multiple respiratory pathogens is common [12-15].

In 2012, an influenza surveillance study in Zambia reported that seasonal influenza and its attributed illnesses were associated with both mild and severe respiratory illness that contributed to outpatient and inpatient respiratory illness, with children less than 5 years of age being the most affected [16]. However, only 128 (4.3%) of 2,976 patients with respiratory illness in 2011–2012 were positive for influenza. As such, it is necessary to broaden detection to include other respiratory pathogens, to estimate the scope of disease burden and determine which respiratory pathogens are commonly identified in SARI patients. There has been little research into non-influenza pathogens identified in SARI patients in Zambia. As a result, little is known about the pathogens involved in severe respiratory infections affecting children under five in Zambia.

To address this challenge, we identified pathogens from hospitalized children under five with SARI in Lusaka, Zambia. The aim of this study was to detect upper respiratory pathogens in specimens that were previously laboratory-confirmed influenza negative, to investigate the relative frequency of isolation, seasonality, and clinical diagnosis of various pathogens identified from SARI patients.

## Methods

### Site and population

From January 2011 to December 2012, we conducted influenza surveillance at a sentinel site at the Pediatrics Department, University Teaching Hospital (UTH) in Lusaka, Zambia. The UTH is Zambia’s largest reference hospital, with a population of over 5,000 children seen in one calendar year; of these, over 2,000 are acute respiratory infection admissions. Zambia has two distinct seasons: the cold season (May to August) and the hot season (September to April). Surveillance was conducted year-around, and patients were drawn from throughout Lusaka.

### Case definitions

We enrolled patients presenting to the UTH with SARI, defined according to a WHO case definition [17] of:

Patient ≤ 5 years of age admitted with less than 7 days duration of illness, with temperature ≥38°C OR history of fever and cough OR sore throat and difficulty breathing.

Hospitalization was a required part of all SARI cases. For each patient, surveillance officers recorded specific signs and symptoms so that case classification could be validated with the case definition form [17].

### Specimen collection

Eligible patients were identified by surveillance officers at the UTH Pediatrics Department, and a WHO questionnaire [17] was used to capture demographic data, clinical information, and influenza vaccination status from parents or guardians representing the children. Written informed consent was obtained from guardians. Combined oropharyngeal (OP) and nasopharyngeal (NP) swabs were collected. A cotton flocked swab was used to rub the back of the OP mucosal membrane for 1–2 seconds, and then placed into 1.5 ml of commercially available Copan Universal Transport medium (UTM) (Copan, Brescia, Italy). A tipped flexible aluminum-shafted NP swab was then inserted into the nose until it reached the nasopharynx, where it was rotated for 1–2 seconds. The NP swab was inserted into the same 1.5 ml vial with UTM as the OP swab. Respiratory specimens were stored at 2–8°C before being transported daily at 4°C from the Pediatric Department to the Virology Laboratory, Department of Microbiology and Pathology, UTH.

### Specimen processing

Specimens with a laboratory confirmed influenza negative result by real-time reverse transcriptase PCR (rRT-PCR) were processed in the current study. The rationale for this was to detect other pathogens other than influenza in children with SARI. The influenza rRT-PCR test was the standard assay from the Centers for Disease Control and Prevention which identifies and subtypes seasonal influenza A/H1N1, seasonal influenza A/H3N2, 2009 pandemic A/H1N1, and influenza B. Specimens were processed only if the internal control human ribonuclease gene had a cycle threshold ≤35, indicating a high-quality specimen [13]. Of the specimens collected during the study period, 4.3% tested positive for influenza; these were excluded from the study. The singleplex assay targeted seven viruses, while the multiplex targeted thirty-three bacteria, fungi, and viruses. Both methods were internally controlled and semi-quantitative. The methods were not directly compared; different sets of samples were used for each method. Therefore, analysis for each method is presented separately.

The singleplex rRT-PCR tests were performed using the 7500 fast real-time PCR system (Applied BioSystems, South Africa) targeting seven viral pathogens: human parainfluenza virus-1, 2, 3 (HPIV), human rhinovirus (HRV), human adenovirus (HADV), human metapneumovirus (HMPV), and respiratory syncytial virus (RSV). Extraction of viral nucleic acid was conducted using a Qiagen body fluid 250 mini extraction kit (Life Technologies, Germany). The PCR protocol, including primers and probes for real-time PCR detection, were from the Centers for Disease Control and Prevention (CDC) in Atlanta, Georgia [13].

The multiplex rRT-PCR was performed with the Fast Track Diagnostic (FTD) kit (Junglinster, Luxembourg). The FTD kit consisted of eight multiplex RT-PCR reactions, targeted 33 respiratory pathogens, and was performed using the 7500 fast real-time PCR system according to the protocol instructions (Fast Track Diagnostics, Luxembourg) [18]. The following viral and bacterial pathogens were FTD targets: Influenza A, B, and C, HPIV 1, 2, 3, and 4, coronaviruses NL63 (cor63), cor 229, cor 43 and HKU 1, HMPV A and B, HRV, RSV A and B, HADV, Enteroviruses (EV), Parechovirus (PV), Bocavirus (HBoV), Cytomegalovirus (CMV), *Pneumocystis jirovecii (PCP), Mycoplasma pneumoniae (Mpneu), Chlamydia pneumoniae (Cpneu), Streptococcus pneumoniae (Spneu), Haemophilus influenzae type B (HIB), Staphylococcus aureus (Saurs), Moraxella catarrhalis (Morax), Bordetella pertussis (Bord), Klebsiella pneumoniae (Kpneu), Legionella species (Legio), Salmonella species (Salm),* and *Haemophilus influenzae* species. Even though this assay has the capability to detect 33 pathogens, not all pathogens were detected from our samples. In particular, in agreement with the influenza-specific rRT-PCR test, all specimens were negative for influenza with the FTD kit. Nucleic acid extraction for multiplex rRT-PCR was conducted with the EasyMAG Respiratory specimen nucleic acid extraction protocol according to the manufacturer’s instructions (EasyMAG 2.0, bioMérieux, Marcy I Etoile, France).

### Data analysis

Data were entered into a Microsoft Access database (Microsoft Corporation, Washington, USA). SPSS version 16 (IBM, New York, USA) and Microsoft Excel (Microsoft Corporation, Washington, USA) were used for analysis of the seasonal distribution and clinical characteristics of the various pathogens. SARI cases with symptom onset of more than 7 days were excluded, to minimize the possibility of false-negative results. The duration of illness of was calculated based on the date of onset of illness to the date of clinical investigation at the sentinel site.

### Ethical approval

Ethical approval was obtained from the University of Zambia Biomedical Research Ethics Committee. Institutional review was waived by CDC because this study was considered to be a non-research public health activity. Written informed consent was obtained from the guardians of all participants for influenza testing, and the University of Zambia Biomedical Research Ethics Committee waived the requirement to re-consent for additional testing.

## Results

### Study population

During the study period, 496 patients who met the SARI case definition and inclusion criteria were enrolled and tested. HIV status of patients was unavailable. The median age of the participants was 9.6 months old. At admission cough was present in 483 (97.4%) of the patients, followed by fever (469; 94.6%) and difficult breathing (399; 80.4%) (Table 1). The most common clinical diagnosis, as determined by attending physicians, was pneumonia (384; 77.4%), followed by bronchiolitis (56; 11.3%). No participant had been vaccinated for influenza prior to being enrolled in this study, and the median number of days between symptom onset and enrolment was 3.75 days (data not shown).

**Table 1:** **Demographic and clinical characteristics of SARI patients (n = 496)**

**Characteristic**	**n (%)**
**Age in years**	
0–2	383 (77.2)
>2	113 (22.7)
**Sex**	
Male	289 (58.3)
Female	206 (41.5)
**Clinical characteristics**	
Cough	483 (97.4)
Fever	469 (94.6)
Difficult breathing	399 (80.4)
Chest in-drawing	188 (37.9)
Sore throat	115 (23.2)
**Clinical diagnosis**	
Pneumonia	384 (77.4)
Bronchiolitis	56 (11.3)
Other	25 (4.0)
Lower RTI	16 (3.2)
Bronchitis	7 (1.4)
TB	7 (1.4)

### Pathogen detection and characterization

One or more pathogens was detected in 381/496 (76.8%) specimens At least one virus was detected from 358/496 (72.2%) of all of the specimens, and at least one bacteria was detected from 197/199 (98.9%) of the specimens tested with the multiplex assay. By singleplex PCR, HRV was the most commonly detected virus (57/297; 19.2%), followed by HADV (50/297; 16.8%), RSV (45/297; 15.2%), and HMPV (14/297; 4.7%) (Table 2). By multiplex PCR, CMV was the most commonly detected virus (415/199; 208.5), followed by RSV (67/199; 33.7%). (Table 2), and the most commonly detected bacteria were *S. pneumoniae* (109/199; 54.8%), *M. catarrhalis* (92/199; 46.2%), and *H. influenzae* (81/199; 40.7%). Using the multiplex assay, single viral infections accounted for 78/199 (39.2%) of cases (Table 2), while multiple infections of viral/viral and bacteria/viral nature were more than the total specimen collected (Table 2).

**Table 2:** **Distribution of viruses among SARI patients using single and multiplex PCR**

**Singleplex PCR (n = 297)**				**Multiplex PCR (n = 199)**			
	**Single infection**	**Co-infection**	**Total**		**Single infection**	**Co-Infection**	
**Viral pathogen**	n (%)*	**n (%)**		**Viral pathogen**	**n (%)**	**n (%)**	**Total**
HRV	34 (11.4)	23 (7.7)	57	HRV	3 (1.5)	45 (22.1)	48
HPIV 1	6 (2.0)	2 (0.7)	8	HPIV 1	0 (0)	15 (7.5)	15
HPIV 2	4 (1.3)	4 (1.3)	8	HPIV 3	21 (0.1)	10 (5.0)	31
HPIV 3	3 (1.3)	1 (0.3)	4	Cor43	1 (0.5)	22 (11.0)	23
RSV	24 (8.1)	23 (7.7)	45	HPIV 4	0 (0.0)	0 (0.0)	0
HADV	27 (9.1)	26 (8.7)	50	RSVA + B	8 (4.0)	59 (29.6)	67
HMPV	11 (3.7)	3 (1.3)	14	CMV	58 (29.1)	357 (179.4)	415
				HADV	4 (2.0)	69 (34.7)	73
				HMPV	0 (0.0)	41 (20.6)	41
				EV/PV	1 (0.5)	12 (6.0)	13
				COR 229	0 (0.0)	0 (0.0)	0
				COR 63	1 (0.5)	22 (11.1)	23
				COR HKU	0 (0.0)	1 (0.0)	1
				HBoV	0 (0.0)	77 (38.7)	77
Total	109 (36.7)	82 (27.6)	184	Total	78 (39.2)	730 (366.8)	808

*The total number of specimens tested by each assay was used as the denominator for each percentage and the total of co-infections is high than total tested because of instances were one virus had multiple infections with other pathogens.

### Seasonal distribution

**Figure 1:**
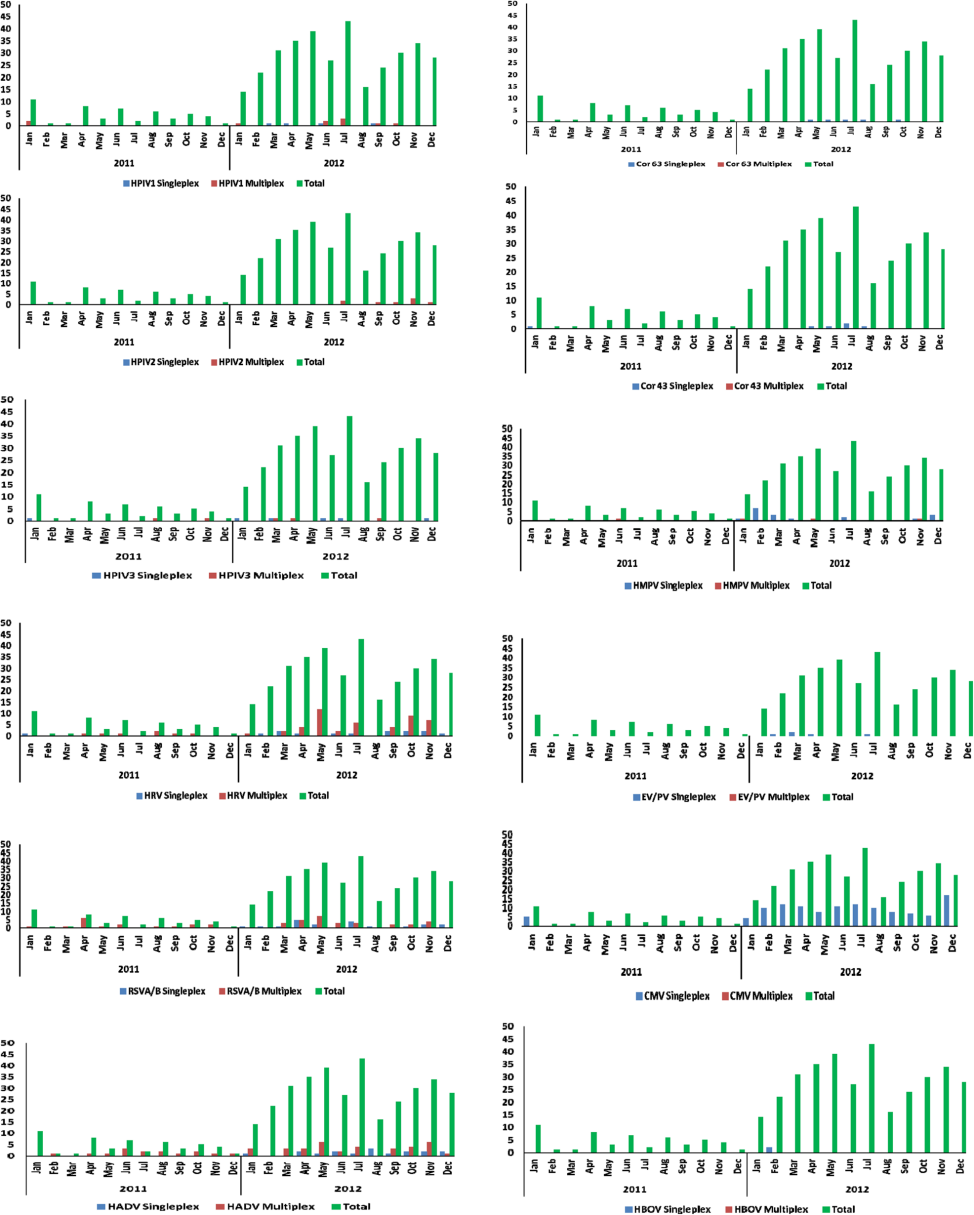
**Seasonality of viruses identified from specimens collected in Lusaka, Zambia from January 2011 to December 2012.** Specimens collected throughout the year were analyzed for seasonality. The total number of specimens collected each month was plotted, along with the number of times each virus was identified per month. The following viruses were monitored: Human parainfluenza viruses 1,2,3 and 4 (HPIV 1, 2, 3 and 4), coronaviruses NL63 (cor63), cor 229, cor 43 and HKU 1, Human metapneumoviruses A and B (HMPV A and B), rhinoviruses, (HRV) Respiratory syncytial viruses A and B (RSV A/B), Adenoviruses (HADV), Enteroviruses (EV), Parechovirus (PV), Bocavirus (HBoV), and Cytomegalovirus (CMV).

**Figure 2:**
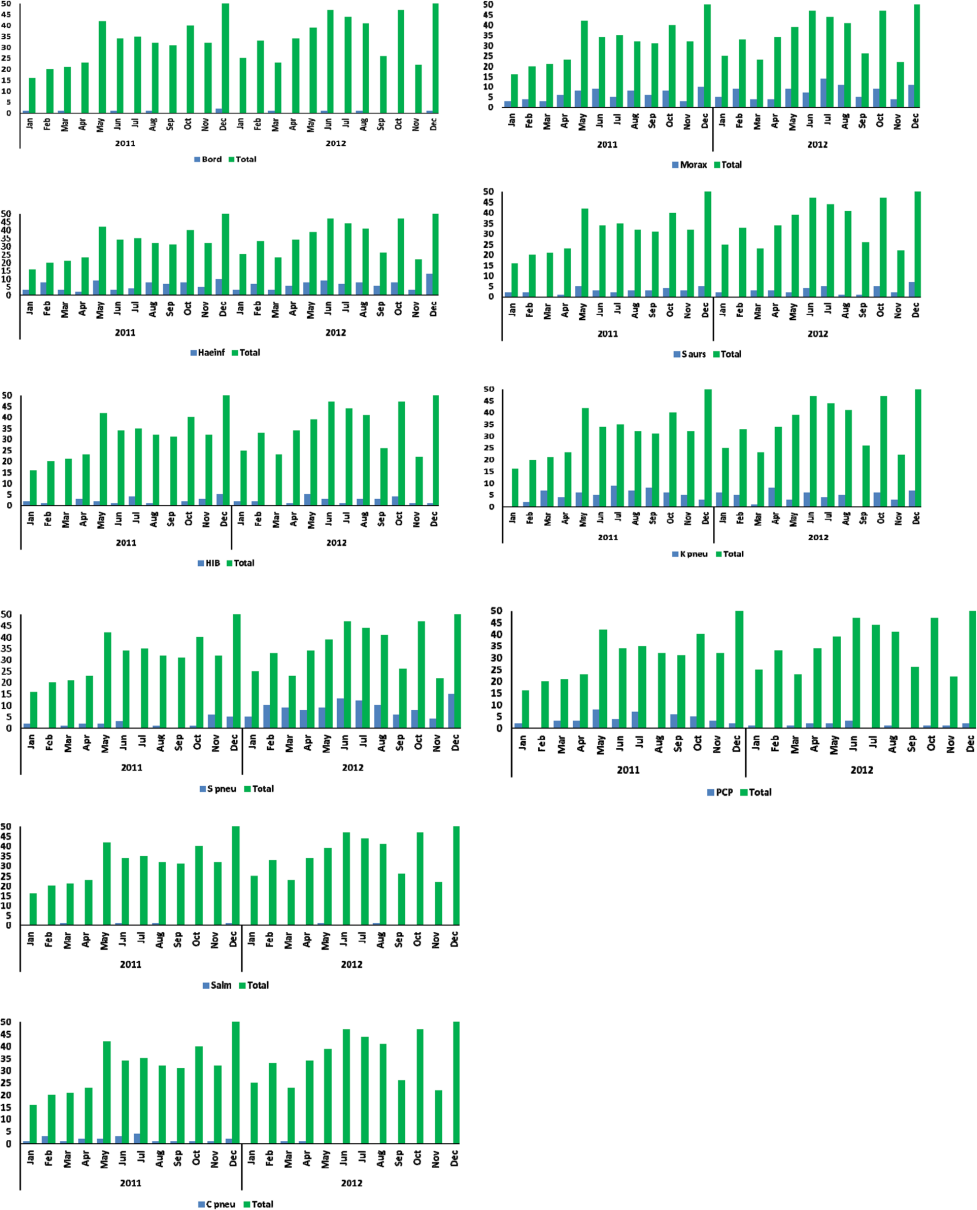
**Seasonality of bacteria/fungi identified from specimens collected in Lusaka, Zambia from January 2011 to December 2012.** Specimens collected throughout the year were analyzed for seasonality. Total numbers of specimens collected each month were plotted, in addition to the number of times each bacteria/fungus was identified per month. The following pathogens were monitored: *Bordetella pertussis (Bord), Haemophilus influenza species (Haeinf), Haemophilus influenzae type B (HIB), Moraxella catarrhalis (Morax), Staphylococcus aureus (Saurs), Klebsiella pneumoniae (Kpneu), Streptococcus pneumoniae (Spneu), Salmonella species (Salm), Chlamydia pneumonia (C pneu), Pneumocystis jirovecii (PCP).*

Zambia has a distinct influenza season, from May to August. In contrast to influenza, most pathogens studied here were detected throughout the year (Figures 1 and 2). RSV was detected year round, with the highest RSV prevalence in April and May. HMPV was most often identified between January and March, which is the rainy season in Zambia. We did not observe a distinct seasonal pattern with the rest of the viruses or with any of the bacteria.

## Discussion

To our knowledge, this study reports for the first time the pathogens detected from many SARI cases in Zambia. Bacterial and viral pathogens were detected from many SARI cases among hospitalized children in Zambia, suggesting that viruses and bacteria are major causes of significant respiratory illnesses [1,2,19].

We detected at least one pathogen in 381/496 (76.8%) samples from SARI cases. A relatively similar pattern was observed in Madagascar, which identified viruses and/or atypical bacteria in 74.6% of 295 samples [20,21]. Viral pathogens were detected in 184/297 (62.0%) of the samples by singleplex and in 167/199 (83.9%) of the samples by multiplex assay. The higher percentage in the multiplex is likely due to CMV, which was the most commonly detected virus with the multiplex but was not a target of the singleplex assay. The high prevalence of CMV in our study is intriguing, and while in the current study we cannot determine if CMV is the cause of SARI in children where CMV was identified, we plan to further examine the potential role of CMV in SARI in the future.

Bacterial pathogens were detected in 197/199 (98.9%) of the samples by the multiplex assay. This prevalence of bacterial detection is comparable to studies done in rural India and among children in Japan, which showed that 90.4% of children carried bacteria pathogens and at least 55.3% had more than one bacterial pathogen [22-24]. According to our study, 119/199(54.8%) of the cases had *S. pneumoniae*, which is relatively comparable to other studies that reported that *S. pneumoniae* was detected from 75.2% of cases [25]. Identification of *S. pneumoniae* likely reflects carriage rather than disease in many patients, but without testing non-SARI controls to determine the proportion positive for *S. pneumoniae*, it is not possible to definitively decide if *S. pneumoniae is* being carried or is causing disease in our study.

We detected viral/viral coinfections in 160/496 (32.3%) patients tested with assays, and bacteria/viral coinfections 167/199 (83.9%) patients tested with the multiplex (Tables 2, 3, and 4). A study in a similar setting in India reported multiple infections in 62.4% of hospitalized children [24]. In Madagascar, dual infections were reported in 27.3% of children hospitalized for acute viral infections [21]. Other studies in resource-limited settings showed comparable results [26-28].

**Table 3:** **Distribution of co-infections of respiratory pathogens in SARI patients using singleplex PCR**

	**HRV**	**HPIV 1**	**HPIV 2**	**HPIV 3**	**HPIV 4**	**RSV**	**HADV**	**HMPV**
**HRV**	X	0	1	1	0	10	11	0
**HPIV 1**	-	X	0	0	0	2	0	0
**HPIV 2**	-	-	X	0	0	0	4	0
**HPIV 3**	-	-	-	X	0	1	0	0
**HPIV 4**	-	-	-	-	X	0	0	0
**RSV**	-	-	-	-	-	X	9	1
**HADV**	-	-	-	-	-	-	X	2
**HMPV**	-	-	-	-	-	-	-	X

**Table 4:** **Distribution of co-infections of respiratory pathogens in SARI patients using multiplex PCR**

	**HRV**	**HPIV 1**	**HPIV 3**	**Cor43**	**RSVA/B**	**CMV**	**HADV**	**HPIV 4**	**EV/PV**	**COR 229**	**HMPV AB**	**COR 63**	**COR HKU**	**HBoV**	**S.aurs**	**C.pneu.**	**S.pneu**	**HIB**	**PCP**	**Legio.**	**Salm.**	**K.pneu.**	**Morax.**	**Haeinf.**	**Bord.**
**HRV**	X	0	0	0	0	9	0	0	1	0	0	0	0	1	2	9	1	3	0	2	0	3	7	6	1
**HPIV 1**	-	X	0	1	1	3	0	0	0	0	0	0	1	1	0	0	1	0	0	0	0	2	3	2	0
**HPIV 3**	-	-	X	0	0	3	0	0	0	0	0	0	0	0	1	0	1	0	1	1	0	1	2	0	0
**Cor43**	-	-	-	X	0	4	0	0	0	0	0	1	0	1	2	0	3	0	1	2	0	1	3	3	0
**RSVA/B**	-	-	-		X	10	2	0	0	0	0	1	0	2	4	0	9	2	2	4	0	4	8	10	0
**CMV**	-	-	-	-	-	X	12	0	3	0	14	4	0	14	25	1	71	13	5	22	1	31	59	50	3
**HADV**	-	-	-	-	-	-	X	0	1	0	0	0	0	3	5	0	11	3	0	3	0	5	13	10	1
**HPIV 4**	-	-	-	-	-	-	-	X	0	0	0	0	0	0	0	0	0	0	0	0	0	0	0	0	0
**EV/PV**	-	-	-	-	-	-	-		X	0	1	0	0	0	0	0	4	0	0	0	0	0	2	0	0
**COR 229**	-	-	-	-	-	-	-	-	-	X	-	0	0	0	0	0	0	0	0	0	0	0	0	0	0
**HMPV AB**	-	-	-	-	-	-	-	-	-	-	X	0	0	2	2	5	4	1	1	1	0	5	4	1	0
**COR 63**	-	-	-	-	-	-	-	-	-	-	-	X	0	0	3	0	3	2	0	1	0	1	3	3	0
**COR HKU**	-	-	-	-	-	-	-	-	-	-	-	-	X	0	0	0	0	0	0	0	0	0	0	0	0
**HBoV**	-	-	-	-	-	-	-	-	-	-	-	-	-	X	3	0	13	3	1	5	0	5	12	11	0
**S.aurs**	-	-	-	-	-	-	-	-	-	-	-	-	-	-	X	0	21	1	6	7	0	10	21	18	1
**C. pneu.**	-	-	-	-	-	-	-	-	-	-	-	-	-	-	-	X	2	0	0	0	0	1	1	1	0
**S.pneu**	-	-	-	-	-	-	-	-	-	-	-	-	-	-	-	-	X	15	7	19	1	22	64	54	3
**HIB**	-	-	-	-	-	-	-	-	-	-	-	-	-	-	-	-	-	X	0	7	1	6	15	18	0
**PCP**	-	-	-	-	-	-	-	-	-	-	-	-	-	-	-	-	-	-	X	1	1	7	6	5	2
**Legio.**	-	-	-	-	-	-	-	-	-	-	-	-	-	-	-	-	-	-	-	X	0	5	14	17	0
**Salm.**	-	-	-	-	-	-	-	-	-	-	-	-	-	-	-	-	-	-	-	-	X	2	2	0	1
**K.pneu.**	-	-	-	-	-	-	-	-	-	-	-	-	-	-	-	-	-	-	-	-	-	X	29	21	1
**Morax.**	-	-	-	-	-	-	-	-	-	-	-	-	-	-	-	-	-	-	-	-	-	-	X	50	3
**Haeinf.**	-	-	-	-	-	-	-	-	-	-	-	-	-	-	-	-	-	-	-	-	-	-	-	X	1
**Bord.**	-	-	-	-	-	-	-	-	-	-	-	-	-	-	-	-	-	-	-	-	-	-	-	-	X

This work, using sensitive RT-PCR assays, emphasizes the need to use different testing methods to identify pathogens from children with SARI in Lusaka. These molecular methods demonstrate the need to continuously monitor and detect new emerging viruses responsible for acute respiratory illnesses. Historically, respiratory illnesses have been associated with influenza virus, respiratory syncytial virus, parainfluenza virus, and adenovirus [29,30]. However, with the development of newer, broad spectrum and sensitive molecular techniques, other viruses in patients with respiratory tract infections are being detected [30]. Our results point to the need to further explore and better understand the role of co-infections in children. We report a high proportion of dual viral/bacterial infections from the multiplex assay 167/199 (83.9%) which calls for careful consideration when associating particular pathogens with specific diseases [31]. Nevertheless, other data from different studies have repeatedly offered evidence indicating virus/bacterial coinfections in the respiratory tract [32-35].

In this study, most pathogens were distributed throughout the year. In Zambia, however, the influenza season is from May to August, and a larger number of specimens are collected during this period compared with other times of year, which may have affected the absolute number of isolations of pathogens in this study during this time. No distinct seasonality was observed for most of the respiratory pathogens, but RSV and HMPV were preferentially detected at particular times of the year. A similar pattern was observed in a study conducted in South Africa, where particular pathogens (RSV and HPIV-3) were detected at different times of year while others like HBoV, HRV and HADV were detected throughout the year [36]. This work has helped us understand the different pathogens that may be responsible for respiratory infections in children.

One limitation of our study is that not all eligible children meeting case definitions were identified and enrolled, because even though the University Teaching Hospital is a reference hospital, patients were free to seek medical attention elsewhere. In addition, we depended on standard questionnaires to capture all data, and in most cases the disease outcome was not known because the surveillance system was not designed to capture all indicators. A related limitation is lack of case follow-up, meaning that the disease severities were not recorded. Also, because influenza negative specimens were tested, co-infection rates among patients infected with influenza virus could not be examined. Most of the specimens were collected in 2012, which means that the seasonality we report was primarily based on 2012. Lastly, this study design did not include non-SARI controls, and as such we are not able to associate pathogen detection with disease severity or clinical diagnosis, and that we cannot determine whether microbiological findings reflect true infection with causative agents, or merely colonization in coincidentally ill children.

## Conclusions

This is the first study of viral and bacterial detections in Zambian children, and we found that viral and bacterial pathogens were detected from many SARI cases among hospitalized children in Zambia. The most commonly identified pathogens were CMV, HADV, RSV, HRV, HMPV, *S. pneumoniae*, and *M. catarrhalis*. These data cannot determine etiologic significance of the pathogens, but they provide an indication of the prevalence of each pathogen in children with SARI. In addition, our study provides a platform for studies that include non-diseased controls to better ascertain etiology and more precisely define the disease burden of various pathogens associated with SARI among children in Zambia.
